# Reporting of oral chemical restraint in the Mental Health Services Monthly Statistics for England

**DOI:** 10.1192/bjb.2025.10

**Published:** 2026-02

**Authors:** Thomas Cranshaw, Harry Matchette-Downes, Keith Reid

**Affiliations:** 1 Cumbria, Northumberland, Tyne and Wear NHS Foundation Trust, Newcastle upon Tyne, UK; 2 Independent Data Scientist, UK

**Keywords:** Data visualisation, epidemiology, human rights, mental health services, register-based epidemiology

## Abstract

**Aims and method:**

This study examines more than 5.8 million bed days of data from private and National Health Service care providers who contribute to the Mental Health Services Monthly Statistics in the UK. The use of oral chemical restraint is compared with provider size, and the relative use of oral chemical restraint as opposed to seclusion is investigated.

**Results:**

The data-set has large amounts of missing data. The use of oral chemical restraint is proportional to provider size in terms of bed days. Analysis of those providers who reliably submit data demonstrates patterns of reported use of oral chemical restraint versus use of seclusion.

**Clinical implications:**

Further research is required into the institutional characteristics that are correlated with increased use of oral chemical restraint. Efforts to investigate the use of restrictive interventions in mental health settings are frustrated by inconsistent reporting.

The coercive use of medication in psychiatry ranges from physical restraint and administration of depot medication to ‘encouragement’ to take oral medication in the context of total effective control of a patient such as occurs in certain in-patient settings. In this paper, we use the term ‘oral chemical restraint’ for consistency with the examined database. Oral chemical restraint refers to the practice of coercive use of oral medication as part of a restrictive intervention. This term is recognised within the mandatory National Health Service (NHS) Mental Health Services Data Set (MHSDS), with healthcare providers in England required to provide data on the use of injectable rapid tranquillisation, injectable non-rapid tranquillisation and oral medication.

The current work aimed to determine the range of reporting practices in the use of oral chemical restraint and to compare this against reported use of seclusion. The intention of measurement of the restrictive use of medication is not to ban it but rather to achieve a reduction in its use in concordance with the World Health Organization public health model.^1^ There is also a desire to see which other changes in restrictive intervention practice may be associated with reductions or increases in medication use as part of a restrictive intervention.

There is a significant national effort underway to reduce the amount of physical restraint used in mental healthcare (https://restraintreductionnetwork.org). It is important that rates of chemical restraint are well understood, as there is a potential unforeseen effect in that efforts to reduce rates of physical restraint may result in unintentional increases in the use of medication. Conversely, it was observed that in Germany, rates of physical restrictions increased during a short period when coercive medication was illegal.^2^ A frequent observation from those with long experience in mental healthcare is that rates of seclusion used to be lower; however, many patients were more sedated, and medication was used more liberally. There is also a clear trend towards the use of atypical antipsychotics, which are associated with less sedation than typicals.^3^

A recent study demonstrated highly heterogeneous reporting practices for restrictive interventions within England, showing that some providers report fewer restraints than expected for their size. Many providers did not report any use of restraint despite being large mental healthcare providers. Funnel plots were used to demonstrate that this is far more likely to represent underreporting than accurate reporting of lower or no use of restraint.^4^ This problem is not restricted to England, and no global definitions of restrictive practice have been adopted.^5^

## Method

### Data collection within the Mental Health Services Monthly Statistics (MHSMS)

All mental healthcare providers are required to submit monthly statistics on the use of restrictive interventions.

Restrictive interventions are defined within the NHS Data Dictionary as deliberate acts on the part of other person(s) that restrict an individual's movement, liberty and/or freedom to act independently in order to:

- take immediate control of a dangerous situation where there is a real possibility of harm to the person or others if no action is undertaken; and
- end or reduce significantly the danger to the person or others; and
- contain or limit the person's freedom for no longer than is necessary.

The NHS Data Dictionary provides a definition of chemical restraint as follows:^6^

‘*The use of medication which is prescribed, and administered (whether orally or by injection) for the purpose of controlling or subduing disturbed/violent behaviour, where it is not prescribed for the treatment of a formally identified physical or mental illness. Any incident recorded as chemical restraint must meet all the criteria of a restrictive intervention*.’

The spirit of the definition appears to be that although some interventions are planned as part of ongoing treatment, there are occasions when the management of an incident entails ‘as needed’ medication which would not otherwise have been given but for the disturbed behaviour.^6^

‘*Notes on chemical restraint Do not record [as needed] PRN medication where it does not meet the criteria for a restrictive intervention*.’

The definition therefore makes a division based on the intentions of those giving the medication, stating that only medication provided with the explicit intent of controlling or subduing disturbed or violent behaviour, and not of treating mental illness, counts as chemical restraint. This may run contrary to the common dual intention of coercive use of medication to both treat mental illness and reduce any associated violent behaviour. In addition, the note excludes p.r.n. medication not given as a restrictive intervention. Early provision of p.r.n. medication is used to manage distress that is anticipated to escalate towards violence, but this may not be recorded as a restrictive intervention.

The definitions rely to some extent on subjective elements and questions of intent. Although definitions are designed to be clear, compliance with reporting may vary from reporter to reporter within a service, and from provider to provider. One example is that some providers may correctly regard a patient being required to stay in their room with the door open as seclusion, whereas others may not. Other factors that may be associated with varied reporting include the use of alternative incident reporting systems, which will have different wording and different prompts for data entry. The wording used within incident reporting systems which feed into the MHSMS does not necessarily map precisely on to the definitions above. However, all providers may be expected to face similar dilemmas. Given the large number of providers, this may be conjectured to ‘even out’ to allow the formation of patterns in the data, for example, that larger hospitals or those with more detention have more use of oral medication as part of a restrictive intervention.^4^

Use of oral medication as part of restrictive interventions is an important measure. As seen in a series of cases from Cumbria, Northumberland, Tyne and Wear NHS Foundation Trust, a large mental health and learning disability NHS trust, reductions in injected medication as part of a restrictive intervention are associated with increases in oral medication.^7^ Analyses in annual reports from that trust by the current authors postulated a positive effect: that some injections were stepped down to oral medication, which in turn was ‘displaced’ towards p.r.n. medication not used in restraint but ‘prophylactically’ or even as more effective regular medication, and in some cases talking therapies obviate the need for any PRN at all. Such processes are simply in keeping with the MHA principle of least restrictive care.

This work develops the methods of previous analysis of the MHSMS (PROD-ALERT) in that it uses seclusion as a purported alternative to as-required medication.^4^ The hypothesis was that services which had less seclusion would use more oral chemical restraint. This was based on reports from senior nurses that there was less seclusion but more medication in the ‘old days’. The increase in seclusion is consistent with apocryphal stories that use of seclusion was seen as a failure and seclusion rooms were used for storage. The decrease in medication is consistent with initiatives such as Stopping Overmedication of People with a Learning Disability and Autism and the reduction in use of high-dose antipsychotics.^8,9^

The data-set used was large. The analysis would have been possible using the Excel technique documented in PROD-ALERT; however, in the present work, Python was used as a more efficient technique to clean the data, calculate statistics and produce graphs. The script and all source data necessary to replicate our study are available online.^10^

The measures used in this study were: MHS76 (number of people subject to restrictive intervention in the reporting period: breakdown by provider: breakdown by intervention type); and MHS24 (number of occupied bed days in reporting period). We also examined whether there was any correlation between use of chemical restraint and physical restraint, based on the hypothesis that there may be a ‘see-saw’ effect by which reductions in physical restraint are related to increases in use of chemical restraint. Oral chemical restraint can be conceptualised as the other side of the ‘restrictive see-saw’ from seclusion.

## Results

We analysed data from 5.8 million bed days from 22 providers over a period of 24 months. This corresponded to 14 225 recorded episodes of use of chemical restraint. The data are summarised in Box 1 and Table 1.

Box 1 Summary statistics for data-set.

Number of monthly reporting periods: 24 (September 2020 to August 2022, inclusive)

Number of providers: 22

Total number of bed days: 5 838 865

Average number of bed days per provider: 265 403

Smallest number of bed days amongst providers: 26 910

Largest number of bed days among providers: 564 230

Range in number of bed days among providers: 537 320

**Table 1:** Summary by provider of chemical restraint data for England for the 24-month period from September 2020 to August 2022, inclusive

Form of chemical restraint	Count	Percentage	Min.	Max.	Mean	s.d.
Oral	5125	36.0	0	2695	233.0	574.9
Injectable rapid tranquillisation	7990	56.2	0	1060	363.2	360.0
Injectable non-rapid tranquillisation	845	5.9	0	240	38.4	64.4
Other (not listed)	265	1.9	0	255	12.0	53.1
Total	14 225	100.0				

The number of interventions using oral chemical restraint was generally proportional to the provider size in terms of bed days. In some months, some providers did not report any oral chemical restraint; these are represented by data points on the horizontal axis.

**Fig. 1:**
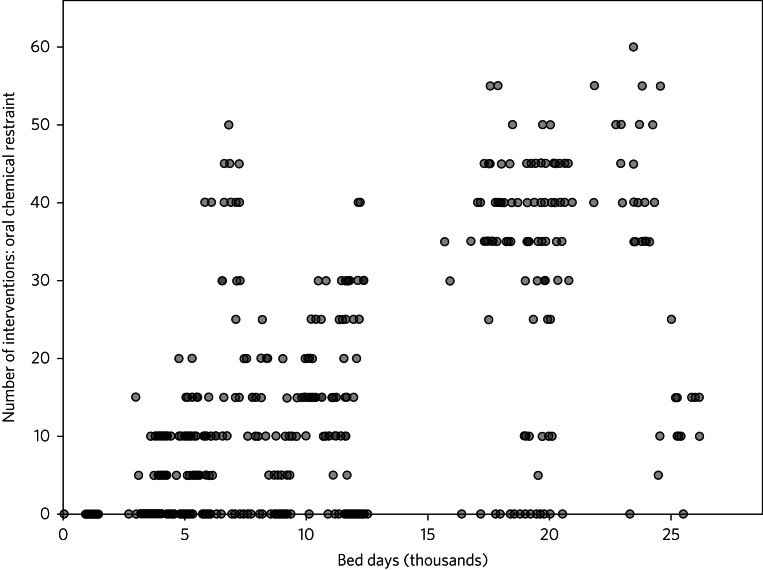
Seclusion rate in comparison with oral chemical restraint rate. There is one point per reporting period (month) per provider. Three large providers are highlighted using shapes. In some months, some providers did not report any oral chemical restraint and/or seclusion; these are represented by data points on the horizontal and vertical axes, respectively.

Figure 1 demonstrates patterns in types of restrictive practice among providers. The number of interventions is expressed per 1000 bed days to account for provider size. Each point represents 1 month of data from one provider. The size of each shape is proportional to the size of the provider in terms of bed days, and the three largest providers are represented by different colours. Radial trends can be seen owing to a rounding feature of the data-set. Rounding to the nearest 5 protects patient anonymity in providers with low numbers of interventions.

Provider A and provider B had similar patterns of reported use of oral chemical restraint versus seclusion. Provider C had a markedly different reporting pattern, with similar use of oral chemical restraint but approximately six-fold lower use of seclusion. Providers clustered along the vertical axis reported relatively high use of seclusion and no use of chemical oral restraint.

**Fig. 2:**
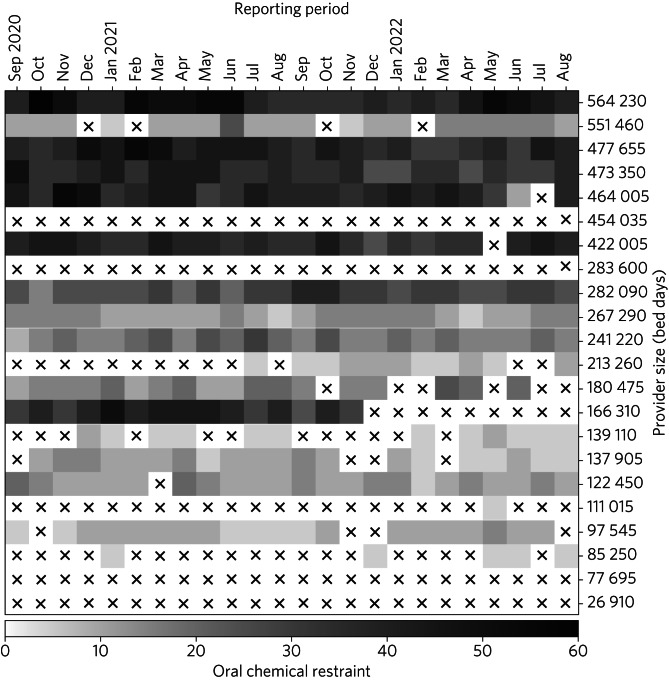
Reporting inconsistencies in oral chemical restraint. Crosses indicate missing data.

Figure 2 illustrates the inconsistent reporting of data on oral chemical restraint to the mandatory NHS MHSDS. Missing data are represented by crosses. Some providers provided no data at all, and many providers were incomplete in their reporting.

## Discussion

**Fig. 3:**
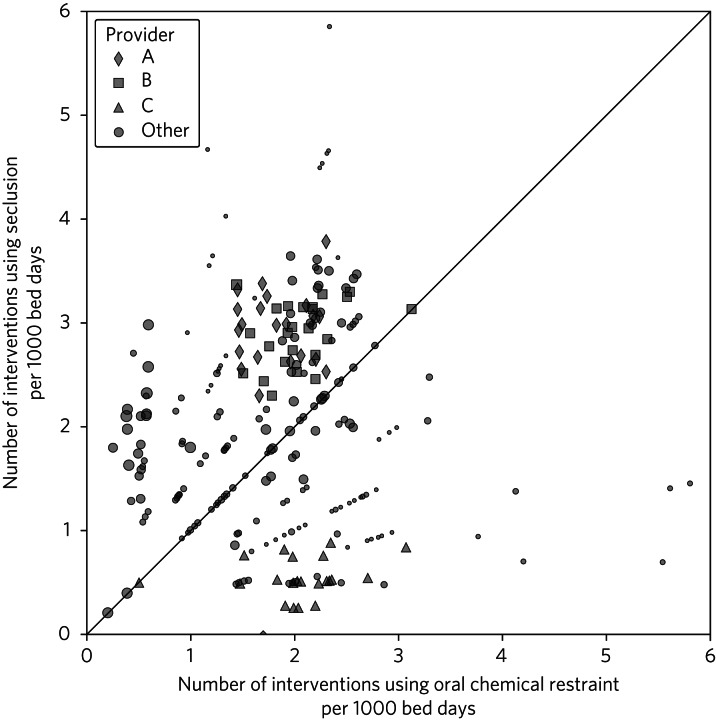
Number of interventions using oral chemical restraint versus number of bed days for each monthly reporting period and each provider.

The analysis of data regarding chemical restraint in England is hampered by inconsistent and improbable reporting similar to that previously described. In Fig. 3, an ‘L sign’ with clustering of data on the y-axis can be seen, as previously documented in restraint data, with many providers reporting greatly lower rates, including no use of chemical restraint at all, than may be reasonably expected for their size.^4^ As shown in Fig. 1, some providers reported similarly improbable use of seclusion without any use whatsoever of oral chemical restraint. In addition, large providers submitted data for some months demonstrating >50 uses of chemical restraint but submitted no data in subsequent months (Fig. 2). A simple measure to improve data reporting would be prompts within incident reporting systems to remind users of the definitions of restrictive interventions. More significant improvements may require pressure on organisations to ensure reliable, accurate, and timely reporting and investigation into the factors underlying missing or implausible reporting.

Contrary to the hypothesis that lower use of medication would be associated with higher use of seclusion, no clear relationship was observed. As shown in Fig. 1, providers did show characteristic patterns in their relative use of oral chemical restraint versus seclusion, with one provider reporting a similar rate of use of oral chemical restraint to that of two other providers but an approximately six-fold lower rate of use of seclusion.

Plausible mechanisms underlying these patterns may include the ‘restrictive see-saw’ or ‘displacement’ of medication. Further analysis beyond the scope of this paper could look for monthly trends for individual providers to see whether there is any evidence of movement between patterns of restrictive practice. Our findings provide some evidence that efforts to reduce certain types of restrictive practice may not necessarily result in compensatory increases in other forms. However, perhaps the most useful practical finding is that public English data on restraint do not yet support detailed analysis of different interventions of the sort we hoped to achieve.

## Data Availability

The original data are available via NHS Data.^11^ The Python script and data used in this research are available via the GitHub repository or on request.^10^

## About the authors

**Thomas Cranshaw**, BA, BMBCh (Oxon), MRCPsych, is a forensic psychiatry registrar at Cumbria, Northumberland, Tyne and Wear NHS Foundation Trust, Newcastle upon Tyne, UK. **Harry Matchette-Downes**, PhD, is a data scientist working independently in the UK. **Keith Reid**, BMedSci(Hons), MBChB, MRCPsych, LLM, AC, PhD, is a consultant forensic psychiatrist at Positive and Safe Care, Cumbria, Northumberland, Tyne and Wear NHS Foundation Trust, Newcastle upon Tyne, UK.
